# Supportive Digital Health Service During Cancer Chemotherapy: Single-Arm Before-and-After Feasibility Study

**DOI:** 10.2196/50550

**Published:** 2023-12-22

**Authors:** Nanna Fridriksdottir, Brynja Ingadottir, Kristin Skuladottir, Sigridur Zoëga, Sigridur Gunnarsdottir

**Affiliations:** 1 Landspitali- The National University Hospital of Iceland Reykjavik Iceland; 2 Faculty of Nursing and Midwifery University of Iceland Reykjavik Iceland; 3 Cancer Registry, The Icelandic Cancer Society Reykjavik Iceland

**Keywords:** web portal for patients with cancer, supportive digital health service, symptom monitoring, self-management support, feasibility, usability, acceptability, patient education, health engagement, patient-reported outcomes, digital health service, patient portal, electronic health records, mobile phone

## Abstract

**Background:**

Digital supportive cancer care is recommended to improve patient outcomes. A portal was designed and embedded within the electronic medical record and public health portal of Iceland, consisting of symptom and needs monitoring, educational material, and messaging.

**Objective:**

This study aims to assess (1) portal feasibility (adoption, engagement, usability, and acceptability), (2) potential predictors of usability and acceptability, and (3) the potential impact of the portal on patient-reported outcomes.

**Methods:**

This was a single-arm, before-and-after feasibility study at a university hospital among patients with cancer who were undergoing chemotherapy. Participation included filling out the Edmonton Symptom Assessment System–Revised (ESASr) weekly and the Distress Thermometer and Problem List (DT&PL) 3 times; reading educational material and messaging; and completing study questionnaires. Clinical and portal engagement data were collected from medical records. Data from patients were collected electronically at baseline and 7 to 10 days after the third chemotherapy round. Usability was assessed using the System Usability Scale (score 0-100), and acceptability was assessed using a 35-item survey (score 1-5). Patient-reported outcome measures included ESASr and DT&PL; a single-item scale for quality of life, family support, and quality of care; and multi-item scales for health literacy (Brief Health Literacy Screener), health engagement (Patient Health Engagement Scale), self-care self-efficacy (Self-Care Self-Efficacy scale), symptom interference (MD Anderson Symptom Inventory), knowledge expectations (Hospital Patients’ Knowledge Expectations), and received knowledge (Hospital Patients’ Received Knowledge). Health care professionals were interviewed regarding portal feasibility.

**Results:**

The portal adoption rate was 72% (103/143), and the portal use rate was 76.7% (79/103) over a mean 8.6 (SD 2.7) weeks. The study completion rate was 67% (69/103). The combined completion rate of the ESASr and DT&PL was 78.4% (685/874). Patients received a mean 41 (SD 13) information leaflets; 33% (26/79) initiated messaging, 73% (58/79) received messages, and 85% (67/79) received follow-up phone calls. The mean System Usability Scale score was 72.3 (SD 14.7), indicating good usability. Usability was predicted by age (β=−.45), ESASr engagement (β=.5), symptom interference (β=.4), and received knowledge (β=.41). The mean acceptability score, 3.97 (SD 0.5), was above average and predicted by age (β=−.31), ESASr engagement (β=.37), symptom interference (β=.60), self-care self-efficacy (β=.37), and received knowledge (β=.41). ESASr scores improved for total symptom distress (*P*=.003; Cohen *d*=0.36), physical symptoms (*P*=.01; Cohen *d*=0.31), and emotional symptoms (*P*=.01; Cohen *d*=0.31). Daily symptom interference increased (*P*=.03; Cohen *d*=0.28), quality of life improved (*P*=.03; Cohen *d*=0.27) and health engagement (*P*=.006; Cohen *d*=0.35) improved, while knowledge expectations decreased (*P*≤.001; Cohen *d*=2.57). Health care professionals were positive toward the portal but called for clearer role delineation and follow-up.

**Conclusions:**

This study supports the feasibility of a support portal and the results indicate the possibility of improving patient outcomes, but further developments are warranted.

## Introduction

### Background

The global burden of cancer continues to grow [1,2], and with improvements in cancer survival, one of the many challenges in cancer care is the integration of evolving supportive care services with the growing population of patients with cancer [3-5]. The need for supportive outpatient services and patient engagement will increase as patients with cancer spend most of their time during cancer treatment outside the health care setting while facing numerous symptoms and self-management challenges. Therefore, to optimize the quality of services, the integration of digital health solutions that provide real-time remote symptom monitoring systems with targeted management functions are considered a crucial part of patient engagement, supportive cancer services, and outcomes [6-10].

Various digital cancer portal solutions have been designed and evaluated with promising outcomes [8,11-14]. These solutions provide functional combinations of symptom tracking and remote monitoring, with or without symptom severity alert systems, and tailored information for symptom and self-management and clinical follow-up, with or without a messaging function to communicate with the health care team (HCT). Digital care options may maximize patient engagement in their care [15], and positive outcomes associated with the regular use of digital patient-reported outcomes (PROs) for symptom tracking and remote monitoring include reduced symptom burden; improved physical function, self-efficacy, and quality of life; reduced cost of follow-up; fewer unscheduled admissions; facilitation of referrals; and improved overall survival [7,8,13,16-20]. Furthermore, but with some inconsistencies, self-care and symptom management digital interventions have been associated with improved informed decision-making and knowledge; better emotional and physical functioning; improved quality of life, self-efficacy, anxiety, and depression; decreased symptom burden; and less distress [8,9,11,12,21-28].

For comprehensive symptom and self-management support, the components of assessment, patient education, and timely feedback are not likely to function without one another, and multicomponent digital interventions including the facilitation of web-based clinical contact and feedback have shown superior outcomes [14]. Other factors that affect and moderate the uptake, engagement, and outcomes of cancer web portals lack consistency and have not been fully explored [14,29]. Provided that there is safe access and health care professional (HCP) endorsement of portals, their uptake and use have been influenced by characteristics such as age, educational level, gender, health status, symptom burden, information accessibility, and level of health literacy and social support [14,29-31]. Furthermore, patients with cancer with lower levels of social support and higher levels of symptom burden have been found to be high users of self-management advice and e-messages via a web-based support system [30]. Moreover, patients with higher symptom burden, lower self-efficacy, higher personal control, and higher health literacy may gain more in terms of symptom reduction and improved quality of life from a comprehensive web self-management intervention [32]. Indeed, depending on the portal design, setting, and target population, web-based portals in cancer care can be feasible and improve patient and system outcomes.

In Iceland, cancer incidence is expected to grow by 49% by 2040 [33], and the national cancer plan recommends the integration of a safe digital support service for patients with cancer and their loved ones [34]. Efforts to develop a digital health service for patients with cancer started as early as 2012 at the Landspitali cancer clinic, which covers >90% of cancer treatments in Iceland. Usual care involves regular treatment visits to the outpatient clinic but limited or no follow-up between appointments. Patients who need support between treatments either make a phone call to the clinic or go to the emergency room if their condition is critical. With the increasing number of patients with cancer and the increasing time restrictions for targeted symptom assessment, patient education, and supportive engagement during encounters, it was decided in 2018 to design and implement a supportive digital cancer portal service.

Informed by cancer web portal research and theories on symptom management and self-efficacy [35,36] and guided by concepts for implementation and feasibility research [37-39], a continuous project was started with the main goal of improving the service and patient outcomes, including patient engagement and symptom and self-care management, and reducing symptom burden during cancer treatment. In late 2021, the implementation of a portal started at the outpatient cancer clinic.

In this paper, we describe both the process of developing the portal and its functions and the results of a feasibility study that was conducted at the outset of implementation in a real-world clinical setting.

### Objectives

The aims of this study were to assess (1) portal feasibility (defined by adoption, engagement, usability, and acceptability), (2) potential predictors of usability and acceptability, and (3) the potential impact of the portal on PROs.

## Methods

### Development of the Cancer Portal

**Figure 1:**
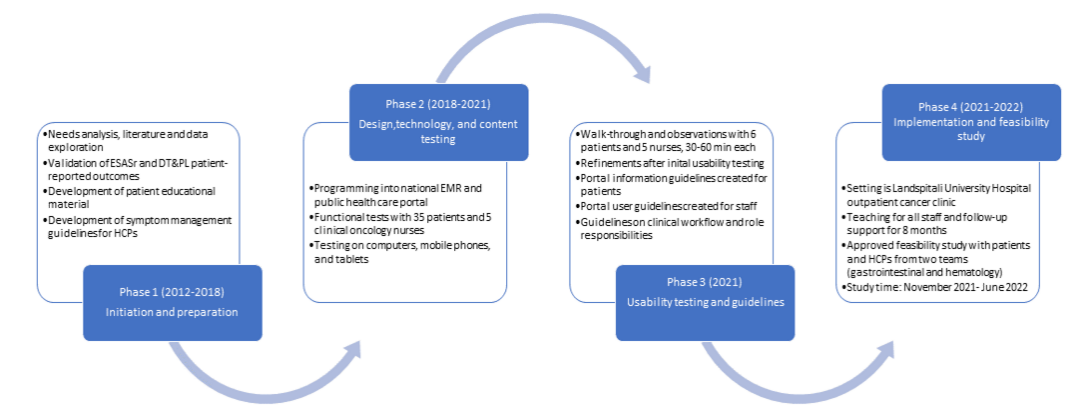
Overview of the timeline and main actions in the 4 developmental phases of the cancer portal. DT&PL: Distress Thermometer and Problem List; EMR: electronic medical record; ESASr: Edmonton Symptom Assessment System–Revised; HCP: health care professional.

On the basis of a needs analysis at the hospital and our prior work on PRO instrument validations [40,41], and in line with the national cancer plan, it was decided in 2018 to start the design and development of the cancer portal. This was done in close collaboration with the Directorate of Health, which is responsible for the national medical records, and users including patients and HCPs and with both financial and tangible support from the Icelandic Cancer Society. A steering committee including main stakeholders was appointed, and a project design group with clinicians, researchers, educators, and IT experts was led by a nurse project manager. The main goal was to develop a user-friendly digital remote symptom monitoring and support system to improve services for patients with cancer through a secure–log-in platform integrated with the existing national electronic medical record (EMR) database in Iceland, which is accessible in both hospital settings and primary care in all health regions in the country. The 4 developmental phases of the cancer portal are shown in Figure 1. The initial work focused on designing and testing 3 main functional components: 1 for monitoring symptoms and needs, 1 for tailored information, and 1 for clinical contact. From 2020 to 2021, extensive technical and user tests were conducted with patients and clinicians, and the functions were improved continuously. The clinical workflow and role responsibilities were defined in close collaboration with clinical administrators. The portal was introduced to the outpatient cancer clinic at Landspitali University Hospital, and over a 6-week period, the staff were informed and trained in groups with individual teaching provided as needed. All 7 cancer treatment teams at the clinic could start using the portal in mid-November 2021, with 2 of them, the gastrointestinal and hematological teams, initiating implementation. The nurse project manager was available for daily support and advice for 8 months.

### Description of the Cancer Portal

The portal is part of the national digital therapeutic health service and is embedded within the EMR system, which can exchange data with relevant systems across the health care system. The staff portal in the EMR provides the primary function of creating groups and an overview for each group and individual. The HCP can filter the patients they are responsible for and mark those they are going to follow up on. The EMR collects dated and timed data with PRO measures, sends reminders, and has a symptom alert system for staff based on defined cutoffs. It makes sending and responding to messages possible as well as managing patient informational material directly and automatically.

Patients have secure access to the portal via a web-based national public health portal that is accessible on mobile phones and computers. An already existing private page is accessible with an electronic ID where citizens have access to basic relevant medical information, parts of their medical record, options for booking appointments, engagement with the primary health care system, overview of immunizations, drug prescriptions, and medication use. The new cancer portal is activated by the hospital HCT in the EMR and provides patients with access and a timed overview of the received patient educational material, instruments for self-assessment, and results from each assessment that they can fill out as requested by the HCP and as often as they want or need. They also have an overview of the messages they send and receive from their HCP.

**Figure 2:**
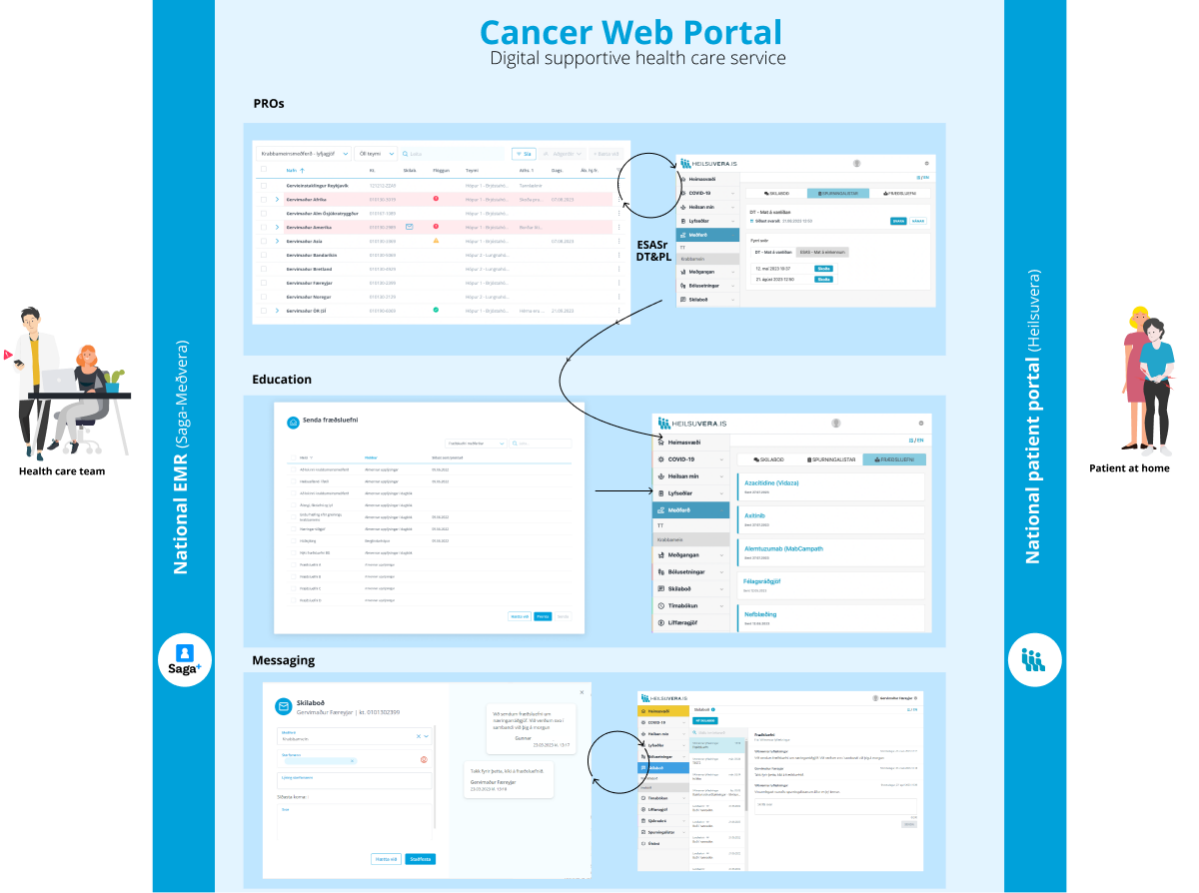
An overview of the portal interface and functions. DT&PL: Distress Thermometer and Problem List; EMR: electronic medical record; ESASr: Edmonton Symptom Assessment System–Revised; PRO: patient-reported outcome.

The interaction between the HCP and patients via the portal leaves data in the patient medical health record, and data on the use of the cancer portal and its PROs are accessible from the hospital data warehouse. Both the EMR and the national health portal are the responsibility of the Directorate of Health and are designed by Origo Healthcare Solutions in collaboration with the main stakeholders and users. The portal includes 3 basic functions. The first is the monitoring of symptoms and needs with 2 widely used and validated PRO measures, the revised Edmonton Symptom Assessment System (ESASr) [40,42,43] and the Distress Thermometer and Problem List (DT&PL) [41,44]. Both measures use an intensity score from 0 to 10, and scores of 0, 1 to 3, 4 to 6, and 7 to 10 are considered as none, mild, moderate, and severe, respectively [40]. In the portal, intensity ratings for ESASr symptoms and distress in the DT&PL are set up for yellow alerts of moderate severity (scores of 3-6) and red alerts of severe intensity (scores of 7-10). All outcomes and alerts are displayed on the EMR patient dashboard, and red alerts also generate an SMS text message to the team nurse coordinator responsible for assessing and responding during the daytime shift via the messaging function or, if necessary, by phone. The second function is targeted information for self-management, which includes a variety of evidence-based patient informational material about cancer treatment, side effects and symptoms, self-care, and services. The HCT has a sorted overview of >200 informational leaflets on the EMR portal with both a printing and sending option. The HCP can tailor and send individual leaflets, group specific information leaflets together, and respond to messages from patients by sending information. Appropriate information leaflets are linked to the symptoms on the ESASr and problems on the DT&PL so that patients receive automated material targeted to the symptoms and problems they identify but they do not receive the same material more than once a month. Finally, the third function is a clinical contact message portal between the patient and the HCT, which is open for short discussions when the portal is activated. All incoming messages from patients are displayed with an envelope on the patient’s dashboard, and the patient receives an SMS text message and an email about having a message from the HCP in the patient portal. An overview of the cancer portal interface and its functions is provided in Figure 2, and a short trailer of the portal can be viewed on Vimeo (Vimeo, Inc; Multimedia Appendix 1).

### Study Design, Sample, and Setting

This was a single-arm study with a before-and-after design suitable for the evaluation of implementation [37]. It had a continuous sample of patients with cancer who had at least 3 planned chemotherapy rounds at the cancer clinic. They had to be aged ≥18 years, understand Icelandic, and be able and willing to use the portal over a period of 3 rounds of chemotherapy.

Between November 23, 2021, and March 1, 2022, patients were recruited from 2 treatment teams (gastrointestinal and hematology). HCPs staffing the 2 treatment teams were sampled to participate in interviews following patient data collection.

### Intervention

Access to the portal was provided to patients after they had signed a written consent form and completed a set of preassessment questionnaires at baseline. Each patient received information guidelines about the portal and received automated reminders once through an SMS text message and email to complete the portal PRO measures (ESASr and DT&PL) at specific time points. The ESASr time points were at baseline, then weekly throughout the study period, and 7 to 10 days after the third chemotherapy round. The DT&PL had 3 time points: baseline, midpoint, and 7 to 10 days following the third chemotherapy round.

At baseline, patients were sent basic informational material about their treatment, side effects, and services (regardless of what they had previously received). In addition, they automatically received informational material based on their answers on the DT&PL and ESASr, but they did not receive the same material more than once a month. They were encouraged to use the message portal to communicate with their HCPs as needed.

The role of the HCPs in the HCTs included one or more of the following actions: registering patients on the patient portal, sending self-assessment forms (ESASr and DT&PL), monitoring and responding to red and yellow symptom alerts, providing patients with relevant additional informational material via the portal, answering messages, or contacting patients via phone who were flagged as red through the symptom alert system or were in need of direct contact.

### Data Collection and Measures

#### Overview

Questionnaires were sent to patients via REDCap (Research Electronic Data Capture; Vanderbilt University) email before they started using the portal and 7 to 10 days after having used the portal over 3 chemotherapy rounds. At baseline, data were collected on personal background and PROs. After the intervention, following portal use after 3 chemotherapy rounds, patients were asked to complete questionnaires on usability, acceptability, and the same PRO measures as at baseline. Data on clinical variables, portal engagement rate, and PROs from the portal ESASr and DT&PL were retrieved from the medical health record and the hospital’s data warehouse.

Data on portal feasibility from the treatment team professionals were collected through semistructured interviews after study completion.

#### Feasibility Measures

##### Adoption and Participation

Patient recruitment, attrition, adoption, and participation rates were logged in Excel (Microsoft Corp). Recruitment refers to the number of eligible patients available during the study period who were offered to participate; attrition refers to the number of patients who were lost (declined vs dropped out at any time during the study). Adoption was defined as the number of patients who intended to use the portal by agreeing to participate and returning the signed informed consent form. Participation rates were based on patients who completed all baseline assessments, used the portal over 3 chemotherapy rounds, and completed the final ESASr and DT&PL. Study completers comprised those who completed all the pre- and poststudy measures.

##### Portal Engagement

The complete portal use rate was defined as the number of patients who used the portal over 3 consecutive chemotherapy rounds and completed the final ESASr and DT&PL self-assessments. Portal engagement rates were based on the following: the completion rate of the assigned portal self-assessment measures, number of red and yellow symptom alerts from the completed self-assessments, number of patients who wanted to talk to an HCP, information leaflet delivery rates, number of portal messages from patients and from nurses to patients, and number of follow-up phone calls from nurses or physicians.

##### Usability

Usability was defined as the perceived usability of the portal. It was assessed using the System Usability Scale (SUS) 7 to 10 days after having used the portal and completed the third chemotherapy round. The SUS is a validated, standardized, 10-item questionnaire measuring the ease of the system in use through agreement with statements on usability for electronic systems, web portals, or devices [45,46]. The agreement scale comprises 5 points, from 1 (“strongly disagree”) to 5 (“strongly agree”), and the total scores are converted to a 0 to 100 scale, providing a global system usability score. A score of ≥68 indicates an above-average usability experience. The internal consistency or Cronbach α in this study was .83.

##### Acceptability

The portal acceptability was assessed using a 35-item survey designed by the authors and 1 open-ended question for comments. Acceptability is a multifaceted concept and refers to the extent to which a health care intervention is judged as suitable, appropriate, favorable, and satisfying [37]. In the design of this survey, we drafted a list of >50 items; discussed which to use; and decided on questions focusing on the perceived general benefits of the portal (10 items), each of the portal functions (14 items), and issues with uptake and use (11 items). We decided to measure the level of agreement on a 5-point scale, from 1 (“strongly disagree”) to 5 (“strongly agree”), and a mean score was computed for the total scale. Agreement rates for those who strongly agreed and agreed were also analyzed for each item. Comments on the open question (“Do you want to add something about the portal?”) were reviewed, similar answers were categorized, and key issues were summarized. Before the study, the survey questions were validated for clarity by 10 patients who had participated previously in the functional testing of the portal, and a few wording adjustments were made. The Cronbach α for the 35-item survey was .87.

#### PRO Measures

##### Portal Patient Outcome Measures

The ESASr includes 9 symptoms: pain, fatigue, drowsiness, nausea, lack of appetite, dyspnea, depression, anxiety, and well-being [40,42]. Each symptom is rated for intensity on a scale of 0 to 10, and the period of assessment is the present. Data from the baseline and postintervention measures were analyzed for a total mean symptom distress score (0-90), total mean physical symptom score (0-60), and total mean emotional symptom score (0-20) and well-being score (0-10) [40,42]. Lower scores are indicative of a better outcome. The validated DT&PL includes a distress intensity scale from 0 to 10 and a list of 40 problems rated as “yes” or “no” for occurrence [41]. The period of assessment is the previous week. Furthermore, patients were asked to report whether they wanted to talk to an HCP (“yes,” “no,” “maybe”). Data from baseline and postintervention measures were analyzed for mean distress intensity, mean number of identified problems, and rate of those who wanted to talk to an HCP (“yes” and “maybe”).

##### Quality of Life, Family Support, and Perceived Quality of Care

The perceived overall quality of life over the previous 4 weeks, perception of family support at the time of illness, and perceived quality of health care in the previous 4 weeks were each assessed using a single-item measure rated on a 5-point Likert scale, with higher scores indicating a better outcome.

##### Health Literacy

The Brief Health Literacy Screener, which has 3 items (“Chew items”), was used to measure health literacy [47,48]. Each item is effective in detecting inadequate health literacy. They inquire about the perceived ability to understand written health information in relation to the illness, ability to read hospital materials without help, and confidence in filling out medical forms. Each item is rated on a 5-point Likert scale. A single score of ≥3 and a sum score of ≥9 indicate inadequate health literacy. In this study, the scores were reversed so that higher scores indicated adequate levels of health literacy. A total mean score was calculated for the 3 questions. The Cronbach α was .63 for the combined items.

##### Health Engagement

The Patient Health Engagement Scale [49,50] was used to assess patients’ psychological readiness and sense of mastery to be active in their own care. It includes 5 ordinal items assessing the engagement position on a continuum for 4 levels of engagement (blackout, arousal, adhesion, and eudaimonia). Each item is rated on a scale of 1 to 7, which is reverted to a scale of 1 to 4, reflecting the aforementioned levels of engagement. Higher scores indicate higher levels of engagement position. The instrument was translated into Icelandic using a back translation method and validated by 10 patients for clarity. The Cronbach α for the scale in this study was .83.

##### Self-Care Self-Efficacy

The 10-item Self-Care Self-Efficacy scale [51] was used to assess patients’ general confidence in their capacity for self-care maintenance and monitoring and managing symptoms. It is rated on a 5-point Likert scale, and scores are transformed to a standardized score from 0 to 100, with higher scores indicating more confidence in self-care. A score of ≥70 indicates acceptable self-care self-efficacy [52]. The Cronbach α for the scale was .88.

##### Symptom Interference

The 6-item interference subscale of the validated MD Anderson Symptom Inventory [53-55] was used to measure symptom-related daily functional impairment. The interference items are assessed on a scale of 0 to 10, with 0 being “did not interfere” and 10 being “interfered completely” in the last 24 hours. The items are symptom interference with walking, activity, working (including housework), relations with other people, enjoyment of life, and mood. The total mean symptom interference score was analyzed. The Cronbach α was .87 in this study.

##### Knowledge Expectations and Received Knowledge

The Hospital Patients’ Knowledge Expectations and Hospital Patients’ Received Knowledge are 2 validated parallel questionnaires [56] that were used to assess the levels of expected and received hospital information. The instruments include 40 items rated on a 5-point Likert agreement scale. Higher scores indicate more expectations and more received knowledge. The questionnaires include 6 dimensions of empowering knowledge—biophysiological, functional, experiential, ethical, social, and financial—but for this study’s purpose, the total mean scores were analyzed. The Cronbach α was .96 for the knowledge expectation total scale and .95 for the received knowledge scale.

##### Interviews With HCPs

Semistructured interviews were conducted via face-to-face conversations by one of the researchers, a clinical nurse specialist with experience in qualitative research and not involved in the team’s patient care or clinical work. An interview guide (Multimedia Appendix 2) was developed by the research group focusing on the experience and attitude of the HCPs toward the portal and its functions and use. The interviews were digitally recorded and transcribed verbatim.

### Data Analysis

#### Quantitative Data

Data were analyzed using the SPSS software for Windows (version 28.0; IBM Corp). Descriptive statistics were used to report patient characteristics and study variables. Continuous variables were reported as means and SDs, and categorical variables were reported as frequencies and percentages. The feasibility means and background variables were compared using independent-sample 2-tailed *t* tests for 2 sample means and ANOVA for >2 sample means. The Pearson correlation was used for analyzing the patterns of association between the raw scores of SUS usability and acceptability and the continuous background and outcome variables. To model the effect of the respondents on the outcome measures of usability and acceptability, we performed a simultaneous (Enter method) multivariate linear regression analysis. Limited by the sample size, we included the main independent variables in each model. We examined the significance test associated with the unstandardized β coefficients (*b*) to identify variables contributing to the variance of the scores while controlling for the remaining predictors in the model and examined the standardized coefficients (β) to compare their importance in the model. The β coefficient effect size estimates are referred to as small (.10-.29), medium (.30-.49), and large (≥.50).

As this was a feasibility study, no power analysis was performed; however, to analyze the differences in pre- and postintervention scores from the patient outcome measures, we chose to use paired-sample *t* tests (2-tailed). The Cohen *d* effect size was used to measure the difference between the 2 means, and similarly, the Cohen *d* coefficients were referred to as small (Cohen *d*=0.2), medium (Cohen *d*=0.5), and large (Cohen *d*=0.8). For practical purposes, the percentage change in the means was also calculated. The Cronbach α was calculated for all questionnaires, and statistical significance was assumed at *P*<.05.

#### Qualitative Data

Data from the interviews with clinicians were analyzed using a directed approach to content analysis [57]. The following categories were chosen for coding beforehand: (1) symptom monitoring and symptom alert system, (2) delivery of patient educational material, and (3) overall assessment of the patient portal and implications for practice.

The interview transcripts were repeatedly read, and the text was categorized according to each of the predefined categories. In total, 2 researchers analyzed the data separately and then discussed the content until agreement was reached, and then a summary for each category was written.

### Ethical Considerations

The study was approved by the National Bioethics Committee (registration VSN 21-135) and the Institutional Research Committee at Landspitali on June 2, 2021. All participants, patients, and HCPs provided written informed consent.

## Results

### Study Participants

**Figure 3:**
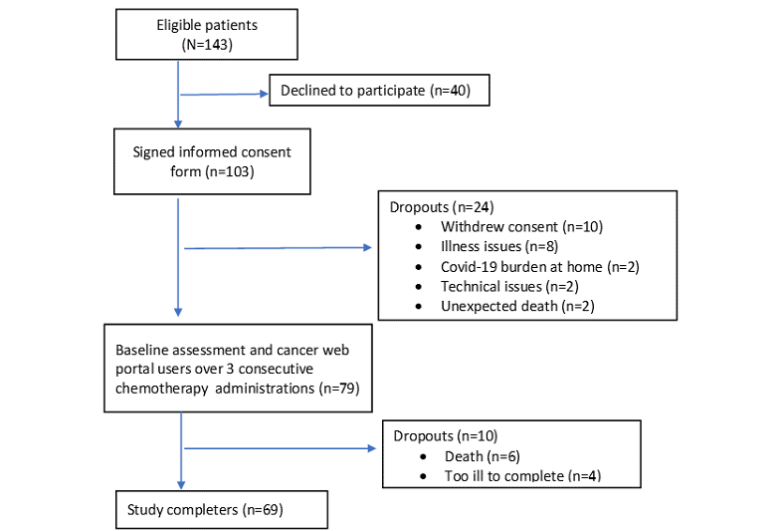
Study flow diagram.

Between November 2021 and June 2022, a total of 143 eligible patients were recruited from the 2 treatment teams who were invited to participate in the study. Of these 143 patients, 40 (28%) declined, and 103 (72%) signed the informed consent forms. Those who agreed to participate were equivalent to those who declined in terms of sex and age. Of these 103 patients, 79 (76.7%) used the portal over 3 chemotherapy rounds, and 69 (67%) finished the study. The dropout rate was 33% (34/103). The patients dropped out at different time points after having signed the consent form, and 62% (21/34) did so because of illness or death. The study flow diagram is shown in Figure 3.

The mean study duration for the portal users over 3 chemotherapy rounds was 8.6 (SD 2.7) weeks, and the background characteristics are shown in Table 1. Their mean age was 61 (SD 13) years, 53% (42/79) were male, and 33% (26/79) had a university education. The mean time since diagnosis was 1.9 (SD 2.9) years, 56% (44/79) had a gastrointestinal cancer, and 46% (36/79) had a documented stage-IV cancer. The vast majority were daily users of smartphones (71/79, 90%), computers (58/79, 73%), and the internet (76/79, 96%).

**Table 1:** Patients’ baseline background and clinical details (N=79).

			Values
**Sex, n (%)**			
	Male	42 (53)	
	Female	37 (47)	
Age (y), mean (SD; range)			61 (13; 21-89)
**Marital status, n (%)**			
	Married or cohabiting	67 (85)	
	Single, divorced, or widowed	12 (15)	
**Highest level of education, n (%)**			
	University	26 (33)	
	High school or secondary school	33 (42)	
	Primary school	20 (25)	
Working outside the home, n (%)			32 (41)
**Residence (n=78), n (%)**			
	Capital area	58 (74)	
	Outside the capital area	20 (26)	
**Daily technology use,** **n (%)**			
	Internet	76 (96)	
	Smartphone	71 (90)	
	Desktop or laptop computer	58 (73)	
	Tablet	43 (54)	
**Cancer type, n (%)**			
	Gastrointestinal	44 (56)	
	Hematological	35 (44)	
Years since diagnosis, mean (SD; range)			1.9 (2.9; 0.02-14.5)
**Stage of cancer, n (%)**			
	I	3 (4)	
	II	11 (14)	
	III	17 (22)	
	IV	36 (46)	
	Missing	12 (15)	
**Other diseases,** **n (%)**			
	None	41 (52)	
	Heart or vascular	11 (14)	
	Diabetes	3 (4)	
	Arthritis	2 (3)	
	Pulmonary	6 (8)	
	Mental	2 (3)	

### Feasibility

#### Adoption and Portal Engagement Rates

The participants’ intention to use the portal or adoption rate was 72% (103/143), and the complete portal use rate over 3 consecutive chemotherapy rounds was 76.7% (79/103).

The patients completed a total of 78.4% (685/874) of portal self-assessments, with a mean of 5.7 (SD 3.8) ESASr and 3.2 (SD 2.4) DT&PL assessments per patient. Adherence to weekly ESASr self-assessments was 70.3% (444/632), and adherence to the DT&PL assessments for the 3 times was 99.6% (241/242). A total of 13.4% (92/685) of the self-assessments triggered a red alarm, and 42.8% (293/685) triggered a yellow alarm. In 30.3% (73/241) of the DT&PL assessments, 52% (41/79) of the patients indicated that they wanted to talk to an HCP (“yes” or “maybe”).

The patients received a total of 3224 information leaflets via the portal, with a mean of 41 (SD 12.9) per patient. Messaging was used by 33% (26/79) of the patients, with a mean of 0.8 (SD 1.1) per patient; nurses sent a total of 101 messages to 73% (58/79) of the patients, with a mean of 1.2 (SD 0.9); and 85% (67/79) of the patients received a total of 178 phone calls from a nurse or a physician, with a mean of 2.2 (SD 2.7). An overview of the descriptive means of portal engagement per patient is provided in Table 2.

**Table 2:** Descriptive mean numbers of portal engagement (N=79).

	Values, mean (SD; range)
ESASr^a^ self-assessments	5.7 (3.8; 0-18)
ESASr red alerts	1.0 (1.1; 0-4)
ESASr yellow alerts	2.7 (2.5; 0-10)
DT&PL^b^ self-assessments	3.2 (2.4; 0-18)
DT&PL red alerts	0.1 (0.4; 0-2)
DT&PL yellow alerts	1.1 (1.0; 0-4)
Wanted to talk to an HCP^c^	0.9 (1.4; 0-8)
Information leaflets received	41 (12.9; 12-79)
Messages from patients	0.8 (1.1; 0-8)
Messages from nurses to patients	1.2 (0.9; 0-8)
Follow-up phone calls	2.2 (2.7; 0-12)

^a^ESASr: Edmonton Symptom Assessment System–Revised.

^b^DT&PL: Distress Thermometer and Problem List.

^c^HCP: health care professional.

#### Usability

The overall mean SUS score for the cancer portal was 72.3 (SD 14.7; range 27.5-100; 95% CI 68.7-75.3; *P*<.001). Compared with the threshold value of 68, the score was statistically significantly higher (t_68_=2.42; *P*=.02), indicating that this portal is above the average usability.

#### Acceptability

The overall mean acceptability score was 3.97 (SD 0.5; range 2.9-4.9; 95% CI 3.87-4.07; *P*<.001). In general, most patients were satisfied with the portal (53/69, 77%), found it personally useful (54/69, 78%), would continue to use it (56/69, 81%), and perceived it to provide them with a sense of security (56/69, 81%). Regarding the portal functions, most patients found the self-assessments easy to use (63/69, 91%) and that conducting the assessments improved their understanding of their own symptoms and needs (53/69, 77%), but 29% (20/69) found weekly self-assessments too frequent. However, 74% (51/69) of the patients perceived that the self-assessments could be helpful for HCPs to monitor the patients’ health, and 54% (37/69) reported that nurses had used information from the portal during their face-to-face encounters. Most participants found the informational material useful (56/69, 81%) and clear (60/69, 87%), and only 12% (8/69) thought that the material they received was too much. Communication via the portal was perceived to be helpful by 86% (59/69) of the participants in dealing with symptoms at home, and 46% (32/69) perceived better quality of care by using the portal. Half (34/69, 49%) of the patients agreed that their loved ones could benefit from having access to part of their health portal or having their own supportive portal.

Only a few participants disagreed with the uptake and use of the portal (4/69, 6%), 4% (3/69) experienced technical problems with the portal, and 5% (4/67) found it distressing to use. In response to the open acceptability question on the survey, 36% (25/69) of participants added comments. All were positive about the portal and its added value to the service, and views for further development and implementation were provided. In Multimedia Appendix 3, the acceptability rates are shown for each survey item by category (portal functions, benefits, uptake, and use). In addition, a summary is provided of the main comments from the patients.

#### Potential Predictors of Portal Usability and Acceptability

The mean SUS usability score correlated strongly with the overall mean acceptability score (*r*=0.652; *P*<.001). The only statistically significant relationship between the mean usability score and background and clinical variables was for age (*r*=−0.346; *P*=.001), indicating less usability with older age. The relationship between the SUS scores and portal engagement variables was significant for the number of ESASr assessments completed (*r*=0.329; *P*=.006) and the number of DT&PL assessments completed (*r*=0.260; *P*=.03), indicating more perceived usability with more self-assessments completed.

The relationship between acceptability and the portal engagement variables was statistically significant for the number of DT&PL assessments completed (*r*=0.265; *P*=.03), indicating more acceptability when more self-assessments were completed. The only statistically significant relationship between acceptability and PROs was for self-care self-efficacy (*r*=0.300; *P*=.04).

To further analyze the practical importance of the findings and the strength and magnitude of the relationship between the dependent variables (usability and acceptability) and possible predictors, we regressed the scores of usability and acceptability on age, engagement with ESASr self-assessment, the symptom intensity of the ESASr physical and emotional symptom scales, daily-life symptom interference, self-care self-efficacy, health literacy, health engagement, and the expected and received knowledge (Table 3). These variables explained 32% of the variance in the usability score and 34% of the variance in the acceptability score. The predictors in this model had small to large effect sizes. Younger age, more ESASr engagement, a higher level of daily symptom interference, and more received knowledge significantly accounted for higher levels of both perceived usability and acceptability. Furthermore, higher levels of self-care self-efficacy accounted significantly for higher acceptability. The standardized β values show which variables in the model had the most influence and how the influential predictors differed in size for usability and acceptability. Engagement with the ESASr self-assessment had more influence on perceived usability (β=.51) than on acceptability (β=.37). Older age had more influence on usability (β=−.45) than on acceptability (β=−.31), whereas daily-life symptom interference, self-care self-efficacy, and having more knowledge had more influence on acceptability than on usability.

**Table 3:** Multivariate regression analysis on the usability and acceptability scores.

	Usability (SUS^a^)^b^						Acceptability^c^						
	b	SE	95% CI	β	*P* value	b			SE		95% CI	β	*P* value
Age (y)	−0.5	0.12	−0.76 to −0.26	−.45	<.001	−0.01		0.01		−0.02 to −0.001		−.31	.02
ESASr^d^ engagement	2.0	0.48	1.05 to 2.97	.51	<.001	0.05		0.02		0.01 to 0.09		.37	.02
ESASr physical intensity	−0.3	0.22	−0.69 to 0.18	−.17	.24	−0.01		0.01		−0.02 to 0.01		−.09	.58
ESASr emotional intensity	0.5	0.55	−0.63 to 1.58	.13	.39	−0.03		0.02		−0.06 to 0.02		−.22	.23
Symptom interference	3.0	1.01	0.97 to 5.03	.43	.004	0.14		0.04		0.06 to 0.22		.60	.001
Self-care self-efficacy	0.2	0.11	−0.06 to 0.38	.19	.15	0.01		0.01		0.001 to 0.02		.37	.04
Health literacy	−0.5	0.92	−2.36 to 1.33	−.07	.58	−0.04		0.04		−0.12 to 0.04		−.15	.33
Health engagement	6.2	3.39	−0.65 to 12.96	.26	.08	−0.03		0.13		–0.28 to 0.23		−.04	.83
Expected knowledge	−0.2	3.3	−8.55 to 4.53	−.07	.54	0.16		0.14		−0.12 to 0.43		.15	.26
Received knowledge	5.23	2.5	0.22 to 10.23	.25	.04	0.29		0.11		0.08 to 0.50		.41	.009

^a^SUS: System Usability Scale.

^b^*F*_10_=4.057; *P*<.001; *R*^2^=0.425; adjusted *R*^2^=0.320.

^c^*F*_10_=3.47; *P*=.003; *R*^2^=0.488, adjusted *R*^2^=0.346.

^d^ESASr: Edmonton Symptom Assessment System–Revised.

### The Perspective of HCPs

#### Overview

In total, 56% (5/9) of the clinicians (staff nurses and physicians) from the 2 treatment teams were available and participated in the individual interviews, all with extensive experience in the care of the patient groups. The interviews lasted between 20 and 40 minutes (mean 28; median 26 min) per participant. Overall, they agreed that the patient portal was an important and excellent addition to patient care, providing opportunities to communicate with patients between treatments, monitor their condition, and deliver written patient educational material in an effortless way. The usability of the patient portal was perceived as good, the system was easy to use, and they did not have any technological difficulties or problems using the portal.

#### Symptom Monitoring and Symptom Alert System

This feature worked well initially and during the study period, but afterward, the participants acknowledged that they forgot to send the questionnaires to the patients and monitor the responses. Checking the alerts and responding to patients was an inconsistent part of their daily practice. They perceived that it was unclear who was responsible for this daily task from day to day even if protocols had been implemented. Another issue had to do with how the alert system was predefined; a red flag was raised for all symptoms at an intensity of ≥7 regardless of the acuity of the symptom or how manageable it was. As an example, fatigue rated as 8 is not surprising, and there are limits to how manageable it is, as opposed to pain rated as 8, which requires immediate attention and is treatable. For this reason, the participants suggested adding a question to the system asking whether the patient wanted or needed assistance with the symptom as patients were reporting their status but not necessarily saying that they expected anything to be done about it. Furthermore, they described how the patients were also experimenting and learning to use the system, and some reported their symptoms frequently—even when they were stable and did not require any response—more for their own surveillance.

#### Delivery of Patient Educational Material

This feature of the portal system was perceived as the best established in daily practice and easy to use. The system provided an appreciated overview of the material sent to patients and had a positive impact as patients came better prepared for their first educational session before the treatment started.

#### Overall Assessment of the Patient Portal and Implications for Practice

Although the overall system as part of the EMR was perceived as both useful and easy to use, the main problems identified by the HCPs had to do with the implementation of this new service. “Everyone helping out” depending on the workload was not working as a strategy; this part of daily work was not prioritized in the busy clinic and was frequently forgotten or left out because of time constraints. The HCPs called for clearer job descriptions, and many pointed out that, as this was an addition to the previous health care services provided by the staff, without being reimbursed or having better staffing, the implementation would not be successful.

### Potential Impact of the Portal on PROs

The paired mean comparisons between the first and final portal self-assessment measures are reported in the first 6 rows of Table 4. For the ESASr, the results showed positive improvements in all symptom scale scores, with small to medium effect sizes and positive percentage changes ranging from 20% to 34%. ESASr total symptom distress scores decreased from 19.4 (SD 13.1) to 15.1 (SD 14.0; Cohen *d*=0.4; *P*=.003), physical symptom intensity scores decreased from 13.8 (SD 9.1) to 11.0 (SD 10.6; Cohen *d*=0.31; *P*=.01), and emotional symptom intensity scores decreased from 2.9 (SD 3.9) to 1.9 (SD 3.1; Cohen *d*=0.31; *P*=.01). For the DT&PL outcomes, the effect sizes were small. The mean distress score decreased by 18%, and the mean number of problems decreased by 6.4%, but the difference was not statistically significant. The proportion of patients who wanted to talk to an HCP identified from the DT&PL decreased from 25% (17/69) to 10% (7/69; *Z*=−2.11; *P*=.04).

**Table 4:** Mean differences in before-and-after outcome measures with estimated effect sizes and percentage change.

Outcome measure (scale range)	Before, mean (SD)	After, mean (SD)	*t* test (*df*)	*P* value (2-tailed)	Effect size (Cohen *d)*	Percentage change
ESASr^a^ total symptom distress score (0-90)	19.4 (13.1)	15.1 (14.0)	3.0 (69)	.003	0.36	22
ESASr physical symptom score (0-60)	13.8 (9.1)	11.0 (10.6)	2.4 (69)	.01	0.31	20
ESASr emotional symptom score (0-20)	2.9 (3.9)	1.9 (3.1)	2.6 (69)	.01	0.31	34
ESASr well-being score (0-10)	2.7 (2.3)	2.1 (2.1)	1.9 (69)	.07	0.22	22
DT&PL^b^ distress score (0-10)	2.8 (2.3)	2.3 (1.8)	1.6 (59)	.11	0.21	18
DT&PL problems (0-40)	8.3 (5.2)	7.8 (4.5)	0.8 (48)	.42	0.12	6
Quality of life (1-5)	3.2 (0.9)	3.5 (0.9)	−2.3 (68)	.03	0.27	9
Family support (1-5)	4.3 (0.9)	4.1 (0.9)	1.4 (68)	.16	0.17	4
Quality of care (1-5)	4.5 (0.6)	4.4 (0.6)	1.4 (68)	.16	0.17	2
Health literacy (0-12)	9.6 (2.1)	10.0 (2.1)	−1.96 (64)	.06	0.24	4
Health engagement (1-4)	3.1 (0.6)	3.3 (0.6)	−2.9 (66)	.006	0.35	6
Self-care self-efficacy (0-100)	68.6 (17.5)	70.7 (17.3)	−1.0 (63)	.31	0.13	3
Daily-life symptom interference (0-10)	2.5 (2.0)	3.1 (1.9)	−2.2 (65)	.03	0.28	24
Knowledge expectations (1-4)	3.5 (0.5)	1.4 (0.5)	20.6 (63)	<.001	2.57	60
Knowledge received (1-4)	2.5 (0.7)	2.4 (0.7)	0.7 (60)	.47	0.09	4

^a^ESASr: Edmonton Symptom Assessment System–Revised.

^b^DT&PL: Distress Thermometer and Problem List.

The results from the paired mean comparisons of the other PROs are reported in rows 7 to 15 in Table 4. The paired difference was statistically significant in 4 of the 9 outcomes. The effect sizes were small to large. The overall quality of life scores improved from 3.2 (SD 0.9) to 3.5 (SD 0.9; Cohen *d*=0.27; *P*=.03), the level of psychological readiness for health engagement improved from 3.1 (SD 0.6) to 3.3 (SD 0.6; Cohen *d*=0.35; *P*=.006), symptom interference with daily living increased from 2.5 (SD 2.0) to 3.1 (1.9; Cohen *d*=0.28; *P*=.03), and knowledge expectations decreased from 3.5 (SD 0.5) to 1.4 (SD 0.5; Cohen *d*=2.57; *P*<.001). The largest positive percentage change was 60% in knowledge expectations.

## Discussion

### Principal Findings

This short-term feasibility study at the outset of portal implementation in a real-world setting supports its overall feasibility from both the patients’ and HCPs’ points of view.

The results suggest that both patients’ adoption rate of the portal and portal engagement were high and that the psychological readiness for health engagement improved significantly. The completion rate of ESASr and DT&PL assessments on the portal was high, and patients perceived the self-assessment tools as easy to fill out and beneficial, not only for themselves but also for their relatives, and the HCP real-time monitoring was highly valued. This finding is very promising and supports the idea that implementing a portal with self-assessment tools is feasible and adds value to patients.

The completion rate of the DT&PL was higher than that of the ESASr, but it is important to keep in mind that participants were only expected to complete the DT&PL 3 times during the study period, whereas they were asked to complete the ESASr weekly. As many participants experienced low symptom burden, this frequent assessment may have been irrelevant for some, and in fact, almost one-third of the participants (20/69, 29%) reported that a weekly symptom assessment was too frequent.

In contrast, many participants reported symptoms frequently, even more often than prompted by the system, regardless of whether they had a high symptom burden. The HCPs commented on this in their interviews (ie, that some patients reported symptoms frequently when they were stable), questioning whether this was necessary. However, some patients may have done so for their own surveillance as a way of keeping a diary of their symptoms, which could further help them in planning activities when they feel well.

The HCPs acknowledged the importance of monitoring patients’ symptoms and needs between treatments. On the basis of the interviews with the HCPs, they were positive toward this function at the start of implementation, but as time passed, they experienced an increased burden associated with the new service and felt that it increased their workload as no changes had been made to the staffing model to account for these additional services. This poses a threat to the reliability of services as some patients reported that their self-assessments did not always receive feedback from HCPs even though their scores were relatively high. This finding may tap into some of the most common barriers among HCPs to introducing eHealth solutions, including workload, role definition, the perception that eHealth solutions undermine face-to-face communication, workflow distribution, alignment with clinical processes, and staff turnover [58]. In this study, HCPs provided important feedback on areas of improvement in workflow, role delineation, and implementation. They called for more clearly defined roles and responsibilities, supporting the findings of Byrd et al [59]. Another important finding is that HCPs felt that receiving frequent alarms in response to high fatigue scores was stressful as they considered that there was little they could do to alleviate this symptom. This will need to be explored further both to make sure that HCPs are knowledgeable about all effective interventions aimed at this frequent and troubling symptom and to set realistic expectations with patients up front.

The most used feature of the portal by HCPs was the sharing of educational material with patients. Patients found the educational material to be both useful and clear. HCPs found it easy to deliver the educational material through the portal and noted that some patients were better prepared for their face-to-face patient educational session at the start of treatment than they had been before the implementation of the web portal. HCPs also found it helpful to have a clear overview of what educational material patients had received. The patient educational material was rated by patients as highly acceptable, and knowledge expectations that were medium-low at baseline decreased significantly, whereas the received knowledge rates did not change. These results are in accordance with a recent systematic review that reported that the delivery of patient education through patient portals is increasing and promising for both patient engagement and health outcomes [60].

The message portal was highly valued by patients, but surprisingly, patients’ use of the messaging system was low as only approximately one-third of the participants (26/79, 33%) used this function during the study. It is possible that simply having access provided benefits to patients regardless of whether they used it as the results from the accessibility survey suggested that patients perceived an increased sense of security having access to health care via the portal. There might also be other explanations; for example, previous research by Hefner et al [61] identified several reasons why patients might limit their use of messaging HCPs despite being given the opportunity. Some encountered challenges in the form of technical barriers, whereas others worried about taking up physicians’ time and were confused about what constituted appropriate “nonurgent” messages. In this study, patients did not report technical barriers as overall they found the portal to be easy to use. However, unfortunately, reasons related to the patient-HCP interaction were not explored in this study, limiting our ability to draw conclusions on those factors. The low use of the messaging system is an important finding; however, this is a perceived barrier among HCPs [59], who often fear that such increased direct access for patients to HCPs will result in a higher workload on their part. Although the messaging system was used by HCPs to communicate with patients, the high percentage of patients who received follow-up phone calls from HCPs suggests that the messaging system may have been underused by HCPs.

Both the usability and acceptability of the portal were above average and indicate that the solution is feasible for clinical use. None of the background variables were significantly associated with the acceptability agreement scores, and age was the only background variable related to usability. This implies that, despite the portal benefits, its contents, and uptake, older age may affect patients’ ability to use it. This is in line with what other researchers have identified [29] and may not be surprising as older patients may experience age-related problems such as cognitive and functional issues affecting their ability to use technical solutions [62]. Patient portals can benefit older patients, but barriers are common, and it is important to include these patients in the design process and consider their training and support needs [63] as well as the direct design of the portal interface [64]. Older patients were engaged in the design and testing of the portal in this study, but it cannot be ruled out that more specific attention to their needs in terms of design may have been necessary and that they may have needed more training and support in using it. If adjustments cannot be made to accommodate older patients, other types of health services need to be provided to this population.

Higher levels of usability and acceptability were significantly associated with increased use of the portal self-assessment tools, indicating their ease of use and value for patients. Furthermore, age, symptom interference, and received knowledge were associated with higher levels of usability and acceptability. This supports the fact that patients who are older have more difficulty using the solution, which has been reported in other studies [31,65]. This also suggests that patients who experience less daily symptom interference may consider themselves to have less use for digital support. This has also been reported in other studies [64]. Similarly, those who considered themselves to be more knowledgeable reported lower levels of usability and acceptability. It is not clear whether this reflects that those with more knowledge did, in fact, find the solution to be less usable and acceptable or whether they did not find that it increased access or added new knowledge to what they already possessed. It is of importance that the mean time since diagnosis among the sample was almost 2 years, so it is likely that many of the patients had both received a lot of patient education and formed relationships with the HCT. Self-care self-efficacy was associated with acceptability but not usability. This may suggest that self-efficacy is not an important attribute when it comes to the perception of ease of portal use but that it is an important variable when it comes to believing that the solution might be of help to the individual. Indeed, the overall quality of care was highly rated both at baseline and after portal use, but in the portal acceptability survey, 47% (32/69) agreed that, by using the portal, they perceived a better quality of their health care. The perception of quality care may mean many things, but as patient portals may positively affect engagement and other patient outcomes that are important to them, patient portals may have the potential to enhance the perception of health care quality.

Multicomponent portals are considered important for improving outcomes [22,66]. Although the design and power of this study did not allow for conclusions about causal relationships, they do provide initial support for the possible effects of web portal use on patient outcomes. The effect sizes may help identify variables for further investigation, but the small sample size in this study limited our ability to detect a statistically significant difference, even if it was present.

The effect sizes for some key variables were medium to large, warranting further exploration of the potential effects of the intervention with a more robust design. The portal PROs (ESASr and DT&PL) indicate a practical significance and support the application of these assessment tools to detect changes in symptom and distress intensity as well as in the number of problems over a brief period during chemotherapy. This supports at least the value of remote symptom reporting using validated instruments, as has been reported in other studies [67].

The end goal of services such as the one assessed in this study is to improve patients’ quality of life and well-being. The patients seemed to benefit significantly in relation to symptom intensity, quality of life, health engagement, and knowledge expectations. However, as this was a single-arm study, we cannot rule out that this may be the effect of other interventions that the patients received during the study period. This effect needs to be assessed further in future studies.

An important end goal for web-based services is to decrease the demand for other services, such as acute and inpatient services. Previous research has supported this effect [6,7,16,20,68], whereas other research has failed to do so [7,11,69]. It was beyond the scope of this feasibility study to assess those effects, which need to be studied further.

The findings of this study may help identify those who benefit from using a portal to receive part of their health care, but even more importantly, they help identify those who are less likely to benefit from the portal and those for whom it adds little. This could thereby help health care providers identify which types of services are most appropriate depending on individual characteristics and make better use of available resources.

### Strengths and Novelty of the Intervention

This portal intervention has numerous strengths. To name a few, it combines into 1 intervention 3 different elements that have all been found to be important [14], namely, regular and systematic symptom assessment connected to an alert system connected to the EMR and monitored by HCPs, individualized evidence-based patient education, and access to a messaging system to interact with HCPs. The portal is embedded within the national patient medical records, which are accessible nationwide and across all levels of health care and is not a stand-alone technical solution. This also opens up the possibility of further developments in integrating cancer care across the continuum, from early detection to survivorship follow-up.

### Limitations and Future Directions

As this was a feasibility study intended to identify areas for improvement, assess usability and acceptability, and assess the initial potential effect, its design was limited. This was a single-arm, before-and-after study with a limited number of participants and over a brief period. Furthermore, the study included 2 types of cancer diagnoses at various stages and cycles of chemotherapy, and therefore, future studies using a longitudinal design to explore changes in outcomes over the course of a disease are needed in patients with similar types of diseases and treatment characteristics. To further test the efficacy of the intervention on patient outcomes, a more rigorous research design needs to be used with a larger number of participants and over a longer period.

### Conclusions

Overall, this short-term study supports the idea that a cancer support portal is a feasible solution for patients receiving cancer treatment. It also provides initial effect sizes on patient outcomes that are encouraging for further implementation and testing of a multicomponent web portal. This effect needs to be tested further over a longer period and, in addition to patient outcomes, include measures of system variables such as the use of resources.

Improvements to the intervention are needed to further its implementation and impact. For further development, particular attention needs to be paid to the specific needs of older individuals. This study also identified important system variables that need to be addressed in future research. New web-based services cannot be thought of as an addition to the existing workflow but, rather, should be thought of as a new type of service that calls for changes in workflow and needs to be staffed accordingly. With the increased demand for cancer care, it is inevitable that patients will have to take more responsibility in their care. This calls for increased access for patients to specialized consultations as they navigate their own care at home. Therefore, it is extremely important to build such services with competent HCPs who have the time to attend to those patients but are not simultaneously expected to take care of patients on an inpatient or outpatient basis. It is also clear from interviews with HCPs that roles, responsibilities, and workflows must be clear and that a dedicated HCP must be responsible for follow-up to ensure that patients receive appropriate care. The fact that the portal is directly connected to the medical record and not a stand-alone feature also makes it feasible as a new service in health care.

## Appendix

Multimedia Appendix 1 Short trailer of the cancer portal.

Multimedia Appendix 2 Interview guide for health care professionals.

Multimedia Appendix 3 Acceptability survey, patient agreement rates, and comments.

## Abbreviations

DT&PL: Distress Thermometer and Problem List
EMR: electronic medical record
ESASr: Edmonton Symptom Assessment System–Revised
HCP: health care professional
HCT: health care team
PRO: patient-reported outcome
REDCap: Research Electronic Data Capture
SUS: System Usability Scale
