# Supplementary material for: Supportive Digital Health Service During Cancer Chemotherapy: Single-Arm Before-and-After Feasibility Study
Source: JMIR Form Res. 2023 Dec 22;7:e50550. doi: 10.2196/50550 (PMC10770793; doi:10.2196/50550)
Supplement: Multimedia Appendix 2 [file formative_v7i1e50550_app2.docx]

 Interview guide with HCPs. Examples of feasibility questions

- What are your thoughts now about the portal as a tool to support patients at home?
- What is your experience of using it?
- What do you think about this method for patient engagement in their own treatment and for you to provide support and care? treatment?
- What was your experience when using the portal:
  - - Monitor symptoms with patients’ self-assessments.
    - Respond to the symptom alert system?
- What was your experience of using the portal to deliver patient education material?
- What do you think are the implications for your practice and the patients’ wellbeing to use the portal?
- What, if anything, needs to be improved?
