# Supplementary material for: Supportive Digital Health Service During Cancer Chemotherapy: Single-Arm Before-and-After Feasibility Study
Source: JMIR Form Res. 2023 Dec 22;7:e50550. doi: 10.2196/50550 (PMC10770793; doi:10.2196/50550)
Supplement: Multimedia Appendix 3 [file formative_v7i1e50550_app3.pptx]

## Slide 1
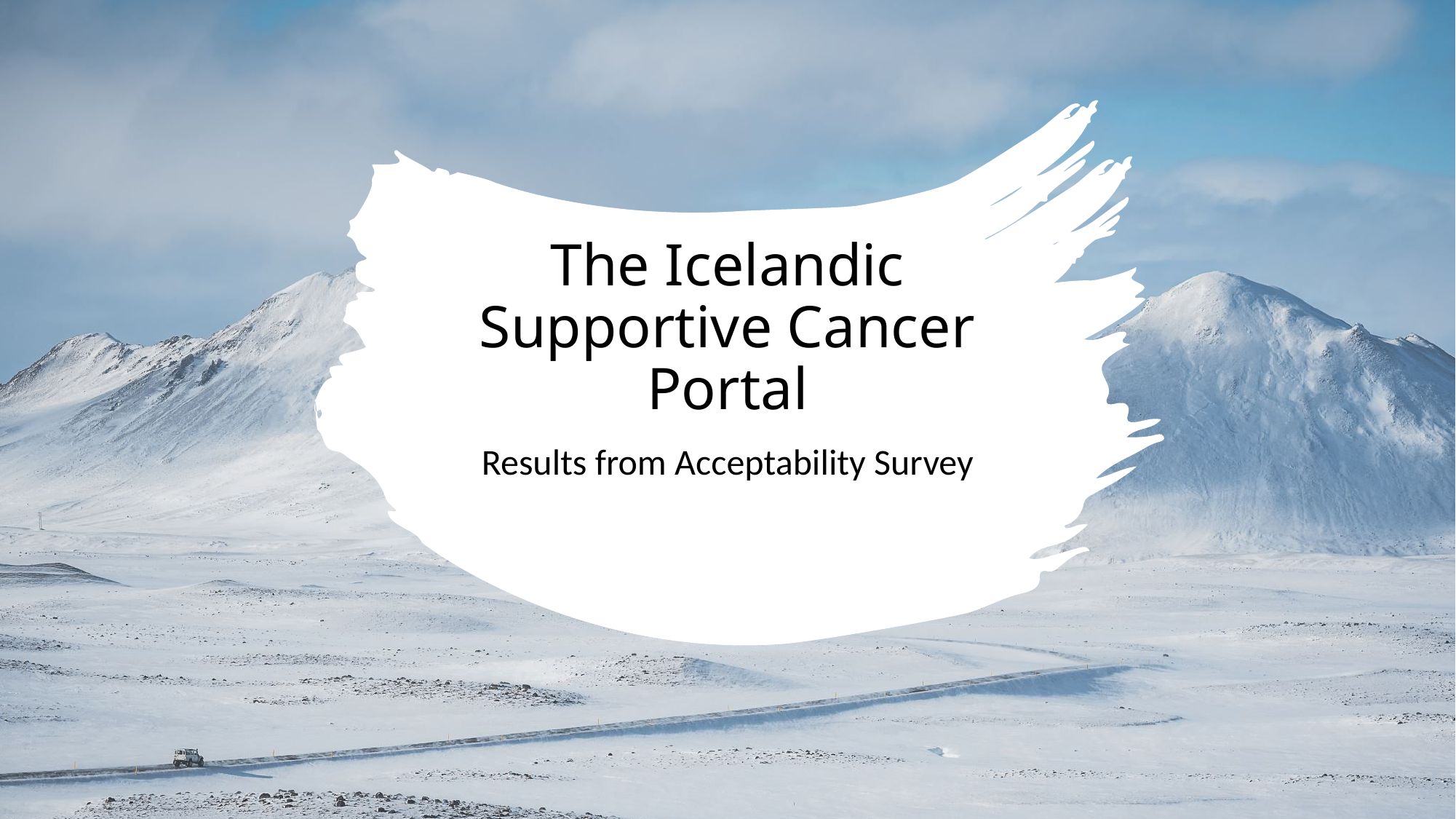

# The Icelandic Supportive Cancer Portal
Results from Acceptability Survey

## Slide 2
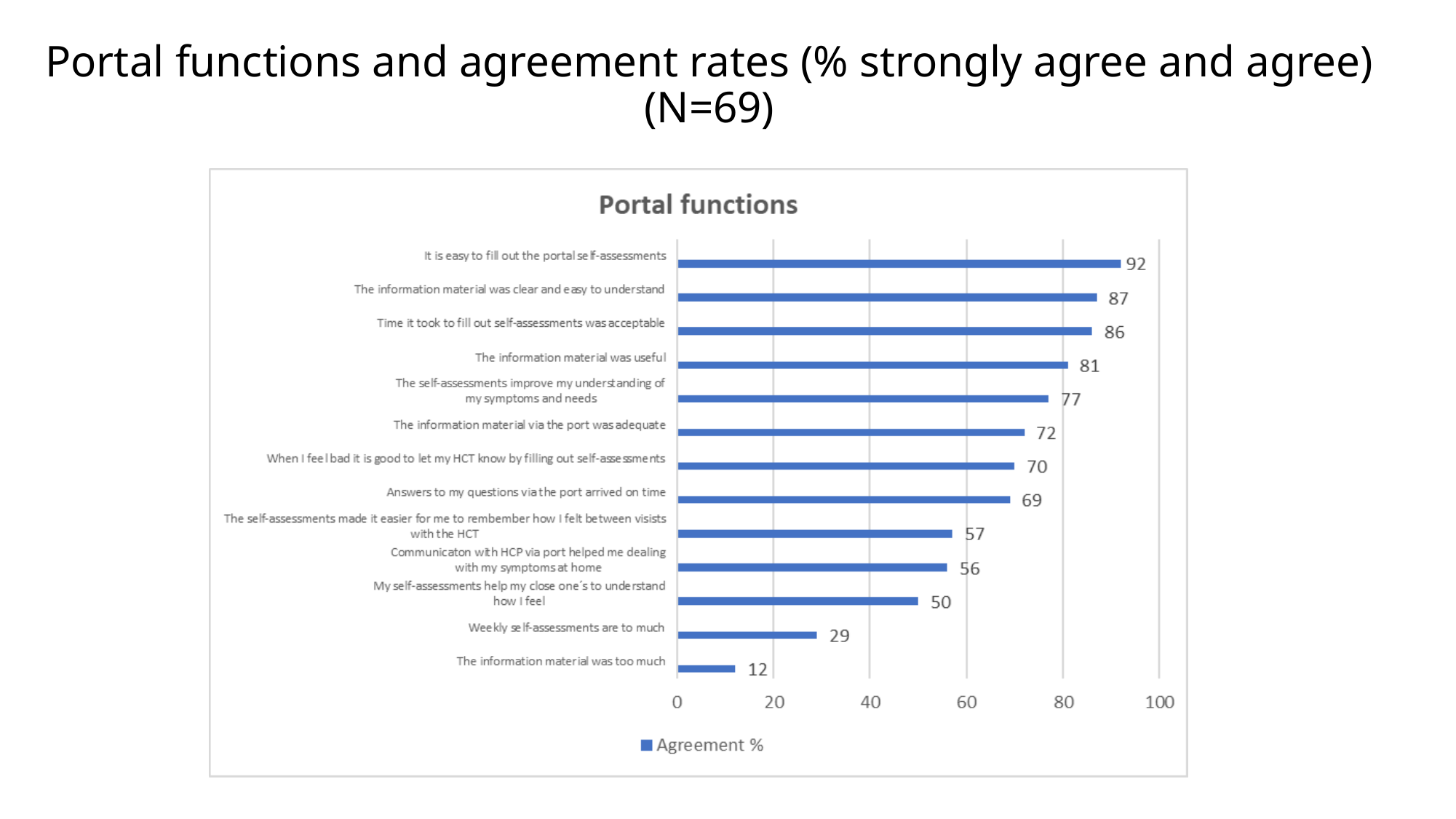

# Portal functions and agreement rates (% strongly agree and agree) (N=69)

## Slide 3
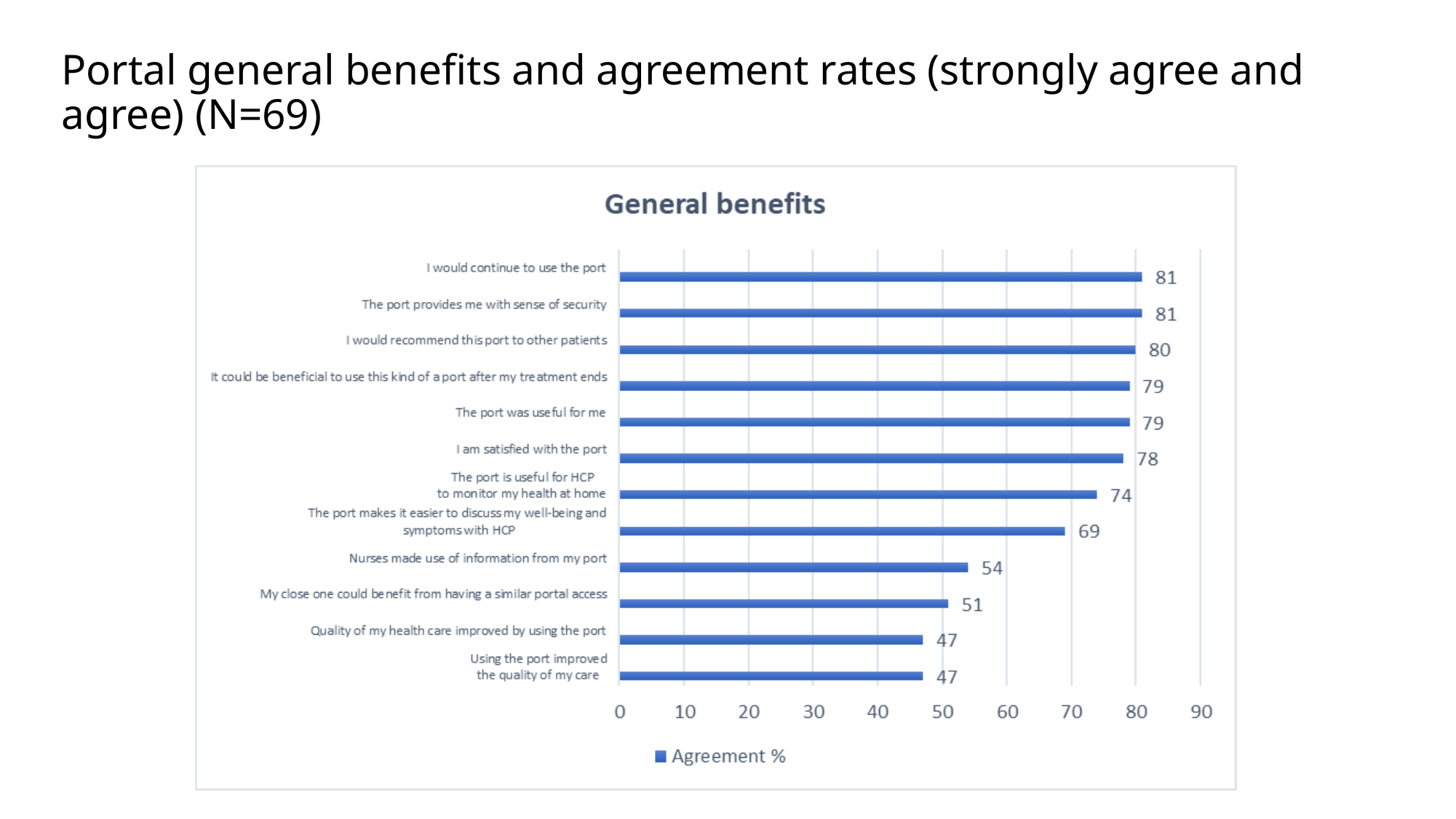

# Portal general benefits and agreement rates (strongly agree and agree) (N=69)

## Slide 4
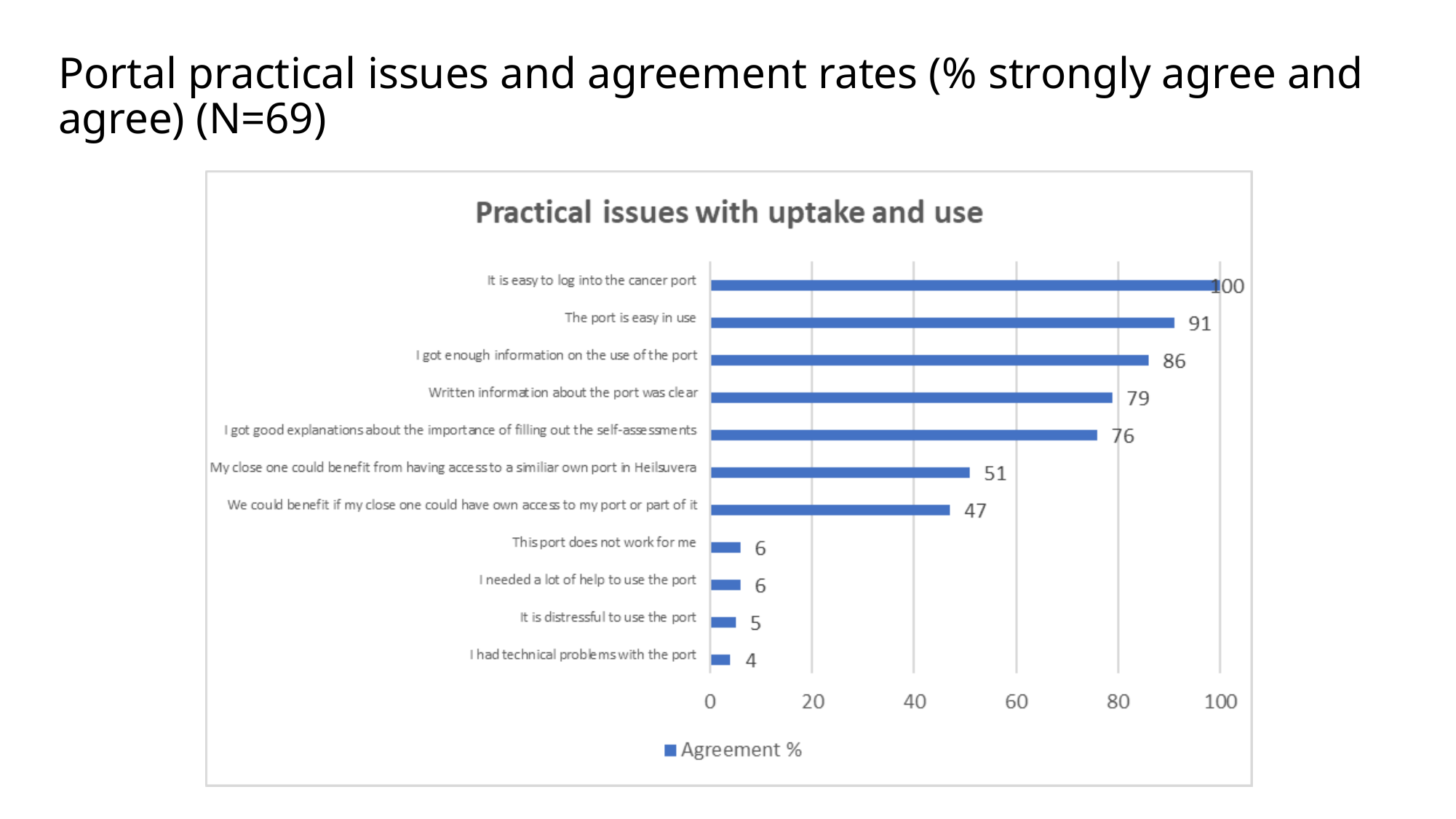

# Portal practical issues and agreement rates (% strongly agree and agree) (N=69)

## Slide 5
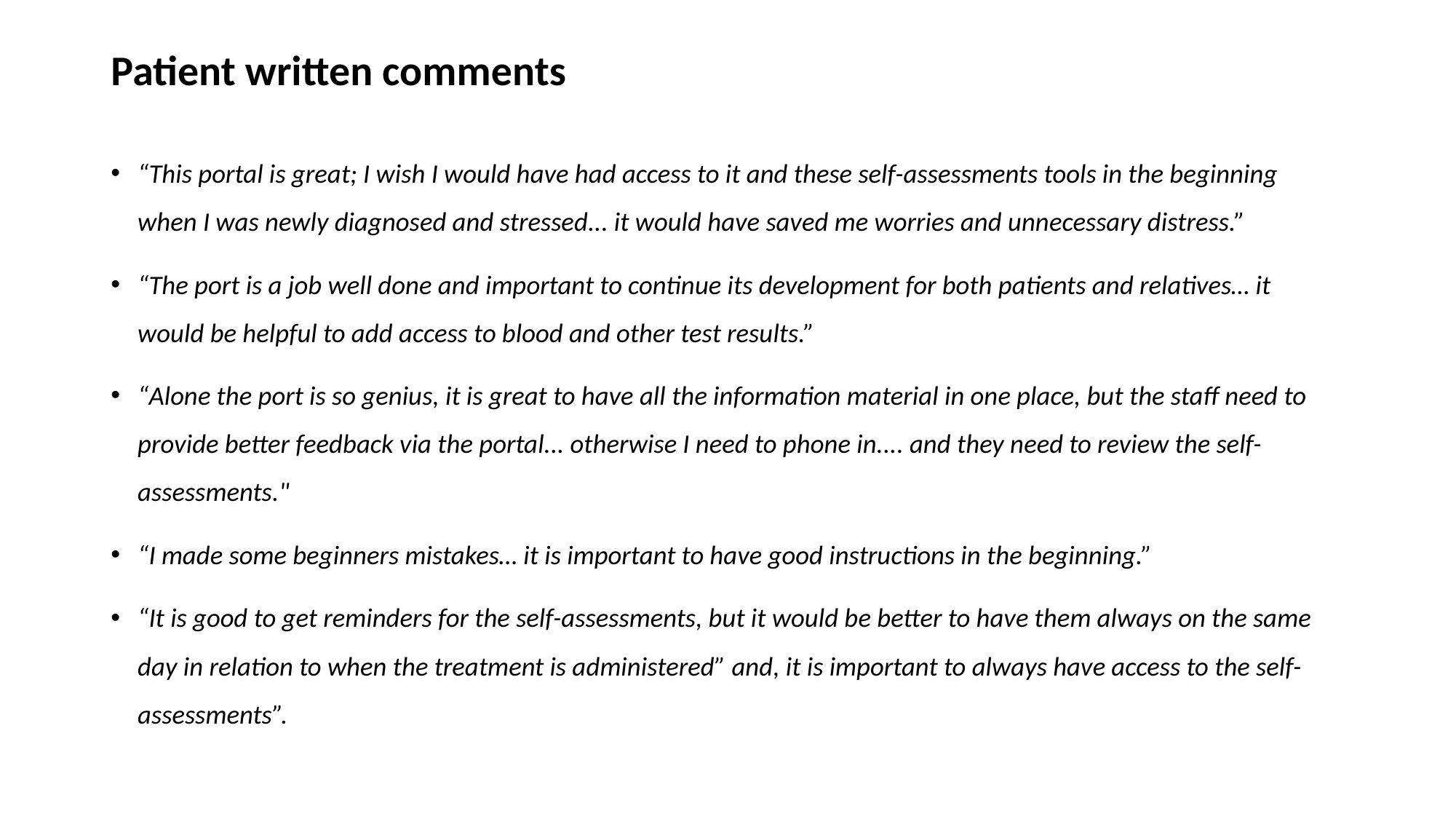

# Patient written comments
“This portal is great; I wish I would have had access to it and these self-assessments tools in the beginning when I was newly diagnosed and stressed... it would have saved me worries and unnecessary distress.”
“The port is a job well done and important to continue its development for both patients and relatives… it would be helpful to add access to blood and other test results.”
“Alone the port is so genius, it is great to have all the information material in one place, but the staff need to provide better feedback via the portal... otherwise I need to phone in.... and they need to review the self-assessments."
“I made some beginners mistakes… it is important to have good instructions in the beginning.”
“It is good to get reminders for the self-assessments, but it would be better to have them always on the same day in relation to when the treatment is administered” and, it is important to always have access to the self-assessments”.
